# Effect of Pulsed Electric Field Technology on the Composition and Bioactive Compounds of Black Soldier Fly Larvae Dried with Convective and Infrared–Convective Methods

**DOI:** 10.3390/molecules28248121

**Published:** 2023-12-15

**Authors:** Radosław Bogusz, Joanna Bryś, Anna Onopiuk, Katarzyna Rybak, Dorota Witrowa-Rajchert, Małgorzata Nowacka

**Affiliations:** 1 Department of Food Engineering and Process Management, Institute of Food Sciences, Warsaw University of Life Sciences—SGGW, Nowoursynowska 159c, 02-776 Warsaw, Poland; radoslaw_bogusz@sggw.edu.pl (R.B.); katarzyna_rybak@sggw.edu.pl (K.R.); 2 Department of Chemistry, Institute of Food Sciences, Warsaw University of Life Sciences—SGGW, Nowoursynowska 159c, 02-776 Warsaw, Poland; joanna_brys@sggw.edu.pl; 3 Department of Technique and Food Development, Institute of Human Nutrition Sciences, Warsaw University of Life Sciences—SGGW, Nowoursynowska 159c, 02-776 Warsaw, Poland; anna_onopiuk@sggw.edu.pl

**Keywords:** edible insects, drying, chemical composition, polyphenols, allergens, fatty acid composition, oxidative stability, thermal properties

## Abstract

In recent years, an increasing interest has been shown in alternative food sources. Many studies are focused on the use of insects. The aim of this study was to investigate the changes in the chemical and thermal properties of black soldier fly larvae influenced by the pulsed electric field (PEF) and convective (CD) or infrared–convective (IR-CD) drying techniques. Examinations of the basic chemical composition, properties of extracted fat (fatty acid composition, acid and peroxide values, and oxidative stability), total polyphenol content, antioxidant activity, allergen content, and thermogravimetric analysis (TGA) were performed. Generally, the results showed that dried black soldier fly larvae are a good source of protein and fat, up to 33% and 44%, respectively. The fat extracted from the dried insects consisted mainly of saturated fatty acids (above 75%), in particular lauric acid (C12:0). A good oxidative stability of the fat was also observed, especially from samples dried with the IR-CD method. The convective drying technique allowed for better preservation of protein content compared to samples dried with the IR-CD method. Nevertheless, samples treated with PEF were characterized by significantly lower protein content. The samples after PEF pretreatment, with an intensity of 20 and 40 kJ/kg and dried with the IR-CD method, were represented by a significantly higher total polyphenol content and antioxidant activity. Furthermore, in most cases, the convectively dried samples were characterized by a higher allergen content, both crustaceans and mollusks. Taking into account all of the investigated properties, it can be stated that the samples without treatment and those that were PEF-treated with an intensity of 40 kJ/kg and dried with the infrared–convective method (IR-CD) were the most rewarding from the nutritional point of view.

## 1. Introduction

The ever-expanding population, potentially surpassing 11 billion in approximately 30 years, consistently drives the exploration for fresh food sources and innovative production methods [1]. The observed need to increase food production to meet the demand for essential nutrients prompts the search for possible solutions. Also, the increasing costs of food production, climate change, environmental pollution, limited freshwater resources, affect this trend [1,2,3]. This trend is related to the pursuit of alternative and sustainable food sources rich in the complete set of proteins needed in the human diet. These sources can include plant-based raw materials (legumes, oilseeds, cereals, and pseudo-cereals), algae and seaweed, microorganisms such as yeast, or bacteria, cell-cultured meat, or edible insects [2,4]. These are sources that not only have comparable or even better nutritional value compared to conventional foods but also generally have fewer negative impacts on the environment.

The potential of insects as an alternative food source is considered one of the most promising directions [5]. Insects are characterized by the presence of many essential nutrients that are needed for human growth and development. They are a good source of complete protein with all the essential amino acids. In addition, insect fat is composed mainly of unsaturated fatty acids, especially oleic and linoleic acids. Carbohydrates are also found in insects, especially chitin, which has properties similar to those of dietary fiber. They also contain essential minerals (e.g., iron, zinc, magnesium, and potassium) and the B-group and the fat-soluble (A, D, E, K) vitamins [6,7,8]. Insects can also contain bioactive compounds, such as bioactive peptides that can inhibit the action of enzymes responsible for the development of the metabolic syndrome [9], or antimicrobial peptides, which could potentially be used in the nutraceutical industry [10].

Among the numerous species, black soldier fly larvae are increasingly being considered as one of the most interesting due to their nutritional value and ability to bioconvert a variety of food waste [11,12]. Black soldier flies, *Hermetia illucens* L. (Diptera: Stratiomyidae) is an insect found in tropical, subtropical, or temperate regions, which actually originated in America. The larvae of this species have a high protein content in the range of 36 to 46% with a desirable amino acid profile, fat in the range of 5 to 49% with a high amount of saturated fatty acids (up to 76%), and chitin in the range of 5 to 8% [11,13,14,15]. Therefore, they could be exploited as a source for biodiesel production [15,16] as well as margarine production [17] or as a potential substitute for other types of fat.

Insects, like most of the other food raw materials, require processing before they can be eaten. Food drying is one of the oldest methods of preservation. The main purpose of drying is to remove water (reduce water activity) from the material and thus prevent microbial growth, reduce enzymatic activity, the oxidation process, or prevent non-enzymatic reactions, thus extending the shelf life of the product with the least possible reduction in quality [18,19]. One of the best-known drying techniques, and the most widely used in the food industry, is convective drying. This process does not require any advanced apparatus or large financial investment but is time- and energy-intensive. However, as a result of the use of high temperatures of the drying air and the long time of exposure of the material, it is considered one of the most destructive drying methods, which causes shrinkage, changes in structure (high hardness) and color, and the loss of many valuable components [20,21,22]. To improve the course of the drying process and the quality of the resulting dried material, various modifications or pretreatment methods can be used, such as infrared radiation (IR) or pulsed electric field (PEF).

Infrared–convective drying uses the effect of electromagnetic waves, most often in the far infrared range (25–100 μm), which are delivered by radiation from heating surfaces to the surface of the material, absorbed by the material, transformed into thermal energy, and then conducted into the material’s interior. The transferred energy induces changes in electrons, causes the vibration of molecules, and thus increases the temperature inside the material relative to the temperature outside, which accelerates heat and mass transfer [23]. The ability of most food components to absorb this radiation can effectively reduce the duration of the process. However, this radiation causes rapid heating of the dried material, especially in the final stage, and can lead to crusting of the material’s surface, which often occurs in drying [24].

Pulsed electric field (PEF) processing is a non-thermal technique based on the application of short electrical pulses of high voltage to a material placed between two electrodes. The application of PEF leads to the electroporation of cell membranes and the formation of pores, by which their permeability increases [25,26]. In addition, the destructive changes occurring in the material due to PEF treatment enable the use of improving unit operations based on heat and mass transfer, such as freezing, drying or extraction [25,27,28,29]. PEF pretreatment before drying facilitates the removal of water from the material during drying, making the process shorter and thus less energy-intensive. As for extraction—increasing the extraction efficiency of an ingredient helps reduce the amount of chemicals used [30]. Thus, this technology is considered more economical and environmentally friendly. Therefore, in this study, the changes in the chemical composition, fat properties, total polyphenol content, antioxidant properties, allergen content, and thermal properties of dried black soldier fly (*Hermetia illucens* L.) larvae affected by pulsed electric field (PEF) pretreatment were investigated.

## 2. Results

### 2.1. Chemical Composition of Black Soldier Fly Larvae Dried with Convective and Infrared–Convective Methods

**Figure 1:**
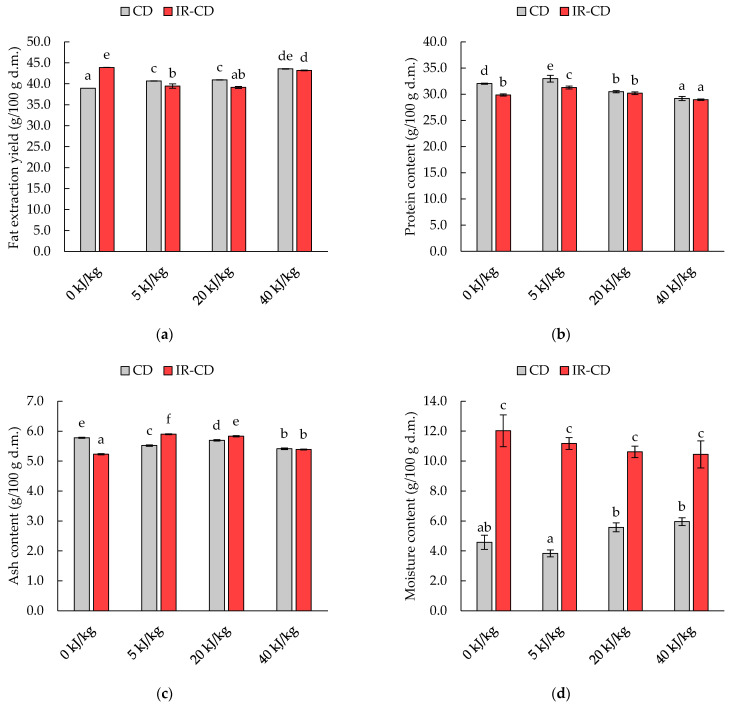
Chemical composition of black soldier fly larvae dried with the convective (CD) and infrared–convective (IR-CD) methods: (**a**) fat extraction yield, (**b**) protein content, (**c**) ash content, (**d**) moisture content. The different letters above the columns indicate significant differences between the samples (Tukey’s HSD, α = 0.05).

Changes in fat extraction yield as well as protein, ash, and moisture content are shown in Figure 1. The most abundant compound in dried black soldier fly larvae was fat (Figure 1a). Statistical analysis (Table 1) showed that the change in the amount of extracted fat from dried insects was influenced by both the intensity of the pulsed electric field (η^2^ = 0.992, *p* < 0.001) and the drying method (η^2^ = 0.724, *p* < 0.001), as well as the interaction between them (η^2^ = 0.992, *p* < 0.001). Analysis of the results revealed that, for dried samples without PEF treatment, a higher extraction yield was seen for the sample dried by the infrared–convective method. On the other hand, in the case of the PEF-treated samples, it was noted that extraction yield increased as the intensity of the PEF treatment increased. Furthermore, for the PEF-treated samples, the convective-dried material was characterized by a significantly (*p* < 0.05) higher fat extraction yield compared to the material dried by the infrared–convective method.

The second compound found in high amounts in the dried black soldier fly larvae was protein (Figure 1b). According to the two-factor analysis of variance, the protein content was influenced by both the intensity of the pulsed electric field (η^2^ = 0.972, *p* < 0.001) and the drying method (η^2^ = 0.897, *p* < 0.001), as well as the interaction between the two (η^2^ = 0.842, *p* < 0.001). Regardless of the pretreatment used, the material obtained by the CD method was characterized by a higher protein content than that obtained by the IR-CD method, with significant differences (*p* < 0.05) found for samples without PEF treatment and treated with PEF at 5 kJ/kg. Moreover, for the sample treated with PEF 5 kJ/kg, the highest protein content was observed. Further increases in the intensity of the electric field of PEF resulted in a significant (*p* < 0.05) decrease in its content.

The ash content varied between the analyzed samples (*p* < 0.05). The two-factor analysis showed that the intensity of the pulsed electric field (η^2^ = 0.986, *p* < 0.001) and the interaction between the electric field intensity and the drying method (η^2^ = 0.989, *p* < 0.001) affected the ash content. For untreated samples, the convective-dried sample was characterized by a higher ash content, while when the PEF intensity was at 5 and 20 kJ/kg, the ash content was significantly higher in samples dried with the infrared–convective method (*p* < 0.05).

The moisture content of the dried insects was influenced by the drying method (η^2^ = 0.976, *p* < 0.001), as well as by the interaction between the intensity of the pulsed electric field and the drying method (η^2^ = 0.664, *p* < 0.001). Significantly higher moisture content (*p* < 0.05) was characterized by samples dried with the infrared–convective method, exhibiting a decreasing trend with increasing intensity of the PEF energy (*p* > 0.05). For samples obtained by the convective method, the lowest water content was found in the material after the application of PEF with an intensity of 5 kJ/kg; 16% lower than that measured in the material without PEF pretreatment. What is interesting, increasing the PEF intensity to 20 and 40 kJ/kg resulted in significantly higher (*p* < 0.05) moisture content.

### 2.2. Fat Properties of Black Soldier Fly Larvae Dried with Convective and Infrared–Convective Methods

#### 2.2.1. Fatty Acid Composition of Fat from Convective- and Infrared–Convective-Dried Black Soldier Fly Larvae

The fatty acid composition of the fat extracted from dried black soldier fly larvae is shown in Table 2. The fat extracted from most of the dried insects was characterized by a higher content of saturated fatty acids (SFA), and a lower content of monounsaturated fatty acids (MUFA) and polyunsaturated fatty acids (PUFA) compared to the fat extracted from the fresh material (see Table 5). The two-factor analysis showed that the intensity of the pulsed electric field (SFA: η^2^ = 0.907, *p* < 0.001; MUFA: η^2^ = 0.836, *p* < 0.001; PUFA: η^2^ = 0.967, *p* < 0.001), the drying method (SFA: η^2^ = 0.979, *p* < 0.001; MUFA: η^2^ = 0.969, *p* < 0.001; PUFA: η^2^ = 0.988, *p* < 0.001), as well as the intensity of the interaction between the intensity of the pulsed electric field and the drying method (SFA: η^2^ = 0.955, *p* < 0.001; MUFA: η^2^ = 0.890, *p* < 0.001; PUFA: η^2^ = 0.985, *p* < 0.001) affected the content of fatty acids from individual groups. The results indicate significant differences in the fatty acid composition between the tested samples. A significantly higher SFA content was noted for the samples dried with the IR-CD method, while MUFA and PUFA content was obtained in a higher amount in the samples dried with the CD method. The SFA group in fat extracted from dried insects was represented mainly by lauric acid (C12:0), palmitic acid (C16:0), and myristic acid (C14:0). The fatty acid from the SFA group found in greatest amount was lauric acid, with a content of 41.56–47.15% and 44.40–47.93%, respectively, for the CD- and IR-CD-dried samples. In the MUFA group, palmitoleic acid (C16:1) and oleic acid (C18:1 n-9c) were found, with a predominant amount of oleic acid. In turn, in the PUFA group, linoleic acid (C18:2 n-6c) and α-linoleic acid (C18:3 n-3) were detected. This group was dominated by linoleic acid (7.55–9.28% and 5.70–7.94%, respectively, for the CD- and IR-CD-dried samples), while the content of α-linoleic acid was in the range of 0.50 to 0.97% (with a significantly higher content of fat extracted from CD-dried insects).

#### 2.2.2. Acid Value, Peroxide Value, and Oxidative Stability of Fat from the Convective- and Infrared–Convective-Dried Black Soldier Fly Larvae

The two-factor analysis showed that the intensity of the pulsed electric field, the drying method, as well as the intensity of the interaction between the intensity of the pulsed electric field and the drying method affected the acid value, the peroxide value, and the oxidative stability (Table 1). The highest acid value was characterized by the fat extracted from fresh insects, whose value was 178.56 mg KOH/g (see Table 5). A significantly higher acid value of insect fat was characterized by samples dried with the IR-CD method compared to those dried using the CD method (Table 3). The acid value increased for the CD-dried samples as the electric field intensity increased, while it decreased for the IR-CD-dried samples. At the highest electric field intensity, in contrast, the values changed their trend—for the CD method the acid value decreased, and for the IR-CD method it increased.

The pretreatments caused a decrease in the peroxide value in comparison to the fat extracted from fresh insects, whose value was 3.48 meq O_2_/kg (see Table 5). Significantly lower peroxide values of insect fat were obtained for samples dried with the IR-CD method compared to those dried using the CD method (Table 3). In the case of CD-dried samples, it was observed that a higher electric field intensity (20 and 40 kJ/kg) resulted in lower peroxide values.

Table 3 shows that the oxidative stability of fat extracted from dried insects, which was measured using the PDSC method and expressed as the induction time, has also been demonstrated. The lowest oxidative stability (22.70 min) was noted for fat extracted from fresh insects (see Table 5). For the dried samples, significantly higher oxidative stability of insect fat was obtained for samples dried with the IR-CD method (Table 3). The changes in oxidative stability were observed to follow the same course as those for the acid value. The oxidative stability increased for the CD-dried samples as the electric field intensity increased, while it decreased for the IR-CD-dried samples; only when the sample was treated with an electric field strength of 40 kJ/kg, was this trend interrupted and changed. The current study showed that the relationships between oxidative stability and total polyphenol content, and between oxidative stability and antioxidant activity, were significant (*p* < 0.05), and equal to r = 0.689 and r = 0.569, respectively.

### 2.3. Chemical Properties of Black Soldier Fly Larvae Dried with the Convective and Infrared–Convective Methods

#### 2.3.1. Total Polyphenol Content (TPC) and Antioxidant Activity (ABTS Assay) of Convective- and Infrared–Convective-Dried Black Soldier Fly Larvae

**Figure 2:**
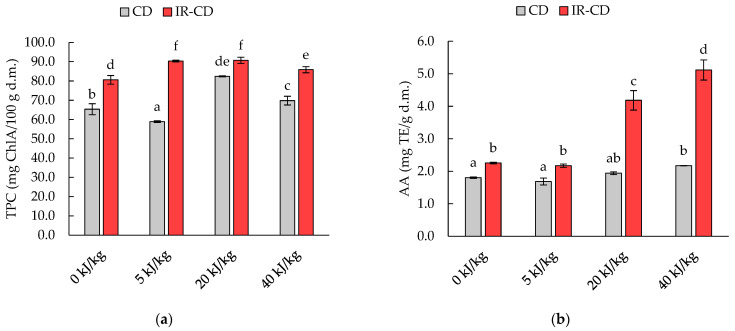
Changes in (**a**) total polyphenol content (TPC) and (**b**) antioxidant activity (AA) of black soldier fly larvae dried with the convective (CD) and infrared–convective (IR-CD) methods. The different letters above the columns indicate significant differences between the samples (Tukey’s HSD, α = 0.05).

The total polyphenol content (TPC) of the untreated and PEF-treated dried black soldier fly larvae is shown in Figure 2a. After drying, the polyphenol content for most samples was significantly higher compared to the fresh material (see Table 5). The TPC depended on the intensity of the pulsed electric field (η^2^ = 0.965, *p* < 0.001), the drying method (η^2^ = 0.987, *p* < 0.001), and the interaction between the intensity of the electric field and the drying method (η^2^ = 0.947, *p* < 0.001). Regardless of the treatment used, the CD-dried material was characterized by a lower TPC (*p* < 0.05) compared to the material dried using the IR-CD method. In contrast, samples treated with PEF before drying had a higher TPC than the untreated material, with the exception of the PEF5_CD sample. It was also observed that increasing the electric field intensity from 5 to 20 kJ/kg increased (*p* < 0.05) the TPC of the CD-dried material, which was not observed (*p* > 0.05) in the IR-CD-dried material. However, a further increase in electric field intensity from 20 to 40 kJ/kg resulted in a decrease in TPC (*p* < 0.05), regardless of the drying method used.

The antioxidant activity of dried black soldier fly larvae, measured against the ABTS^•+^ radical, is presented in Figure 2b. The antioxidant activity was higher than that for fresh material (see Table 5). The antioxidant activity of the dried samples depended on both the intensity of the pulsed electric field (η^2^ = 0.984, *p* < 0.001) and the drying method (η^2^ = 0.986, *p* < 0.001), as well as the interaction between the intensity of the electric field and drying method (η^2^ = 0.973, *p* < 0.001). Lower (*p* < 0.05) antioxidant activity was measured for the CD-dried samples, which, similarly to TPC, is related to the drying temperature. In general, there was no trend linked to the effect of the intensity of the pulsed electric field on the antioxidant activity. However, the application of PEF at 40 kJ/kg for CD-dried insects and PEF treatment at 20 and 40 kJ/kg for IR-CD-dried insects resulted in an increase (*p* < 0.05) in the antioxidant activity.

The current study showed that the relationship between antioxidant activity and total polyphenol content, and between antioxidant activity and protein content were significant (*p* < 0.05), and equal to r = 0.587 and r = −0.598, respectively.

#### 2.3.2. Allergen Content of Convective- and Infrared–Convective-Dried Black Soldier Fly Larvae

**Figure 3:**
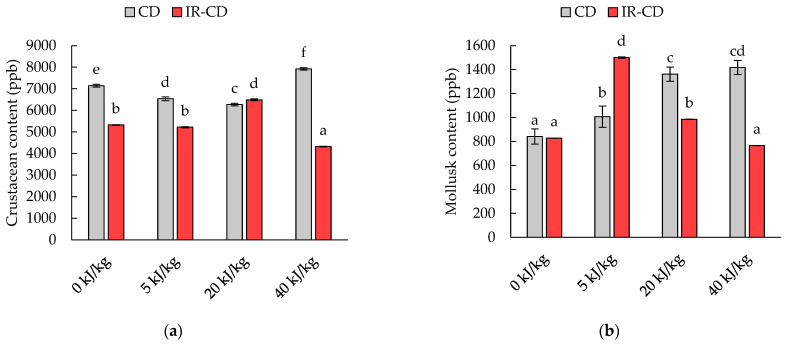
Changes in (**a**) the crustacean content and (**b**) the mollusk content of black soldier fly larvae dried with the convective (CD) and infrared–convective (IR-CD) methods. The different letters above the columns indicate significant differences between the samples (Tukey’s HSD, α = 0.05).

The allergen content of dried black soldier fly larvae is presented in Figure 3a,b. The crustacean content depended on the intensity of the pulsed electric field (η^2^ = 0.914, *p* < 0.001), the drying method (η^2^ = 0.995, *p* < 0.001), and the interaction between the intensity of the electric field and the drying method (η^2^ = 0.993, *p* < 0.001). A significantly higher crustacean content was determined in the samples dried with the CD method compared to the samples dried with the IR-CD method (Figure 3a). Samples treated with PEF at 5 and 20 kJ/kg were characterized by a significantly lower crustacean content compared to the untreated sample, while the sample treated with PEF at 40 kJ/kg contained the highest amount of crustacean tropomyosin. In turn, for samples dried with the IR-CD method, the utilization of PEF at 20 kJ/kg increased, while at 40 kJ/kg the crustacean content decreased compared to the untreated sample. 

The mollusk content depended on the intensity of the pulsed electric field (η^2^ = 0.957, *p* < 0.001), the drying method (η^2^ = 0.811, *p* < 0.001), and the interaction between the intensity of the electric field and the drying method (η^2^ = 0.977, *p* < 0.001). In the case of the CD-dried samples, it was observed that the mollusk content increased with an increasing electric field intensity. In turn, for the IR-CD-dried samples, the content of mollusk tropomyosin increased when the PEF was applied at 5 kJ/kg, and then decreased when the material was treated at 20 kJ/kg. Moreover, for a sample treated with an electric field strength of 40 kJ/kg, the mollusk content decreased more and reached a value similar to the untreated sample.

### 2.4. Thermal Properties of Black Soldier Fly Larvae Dried with the Convective and Infrared–Convective Methods

The results of the thermogravimetric analysis of dried black soldier fly larvae are shown in Table 4. The thermal decomposition of the dried samples occurred in three stages. In the first stage (30–110 °C), the weight loss of the samples was the smallest (in the range of 0.8–1.4%) due to the low moisture content of the samples (3.8–12.0%). In the second stage (110–420 °C), the weight loss of the samples was the highest, from 62.8% to 64.2% for the CD-dried samples and from 61.6% to 76.2% for the IR-CD-dried samples. In the third stage (420–600 °C), the weight loss was greater than in the first stage and was associated with further thermal decomposition of the compounds. What is interesting is that the highest weight loss in the second stage (76.2%) was recorded for the PEF0_IR-CD sample, which was probably related to the limited denaturation of protein (due to the lack of PEF treatment and the low drying temperature) and high fat content (Figure 1).

## 3. Discussion

The chemical composition of insects seems to be one of the most important properties, taking into account the possibility of them being used as an alternative food source [31,32]. The differences in the content of the investigated compounds of dried black soldier fly larvae can be explained by both the material used and the processing carried out. Insect larvae are a very complex biological matrix, and their composition can be related to the stage of development and the quantity of compounds accumulated from feed [32]. The utilization of PEF pretreatment and drying also contributed to changes in the content of individual compounds. The structural changes in the tissue due to the electroporation may have resulted in better solvent penetration into the material and improved the fat extraction yield [28,30], which was proved in a current study. A different effect of PEF application was reported by Alles et al. [16], who studied convective-dried black soldier fly larvae and Bogusz et al. [33] in previous study on freeze-dried black soldier fly and yellow mealworm larvae. These studies did not show any improvement in the oil extraction yield. Moreover, during the application of PEF, free radicals and reactive oxygen species (ROS) can form, which can promote oxidation processes [34]. In addition, because of the open structure, a certain amount of protein may dissolve in water (the medium in which the PEF treatment was performed), thus lowering its content. Protein content was also related to the drying method. The infrared radiation was very well absorbed by the material. This can cause the disruption of secondary and tertiary structure bonds of protein, leading to its denaturation [35,36]. Hence, insects dried with the IR-CD method were characterized by a lower protein content.

Fat can be used as an ingredient in many different products, which is why its properties and quality are so important. The fat properties depend on the composition of fatty acids as well as their chemical structure in the triacylglycerol (TAG) molecule [37]. Similar results to those in the current study are found in the literature. In the study carried out by Mai et al. [38] on black soldier fly larvae, comparable amounts of lauric acid (C12:0), oleic acid (C18:1 n-9c), and linoleic acid (C18:2 n-6c) at levels of 31.9%, 20.2% and 13.0%, respectively, were found. However, the obtained fat was characterized by a higher content of unsaturated fatty acids compared to samples of fat in this study. Slightly different results were demonstrated by Kim et al. [39] in the fat extracted from black soldier fly larvae. They demonstrated a lower content of SFA (65.0%) and a higher content of MUFA (20.6%) and PUFA (14.4%). Nevertheless, the amounts of oleic acid (C18:1 n-9c) and linoleic acid (C18:2 n-6c) were similar to our results. The effect of PEF pretreatment on the fatty acid composition was also investigated in the literature. The study conducted by Alles et al. [16] showed that the obtained oils from convective-dried black soldier fly larvae were characterized by comparable contents of lauric (C12:0), oleic (C18:1 n-9c), and linoleic (C18:2 n-6c) acids. They observed that after the utilization of PEF with a higher electric field intensity, the amounts of oleic and linoleic acids increased significantly. These results are in agreement with those noted in our study for samples dried with the IR-CD method. A possible explanation could be the shorter time needed to dry the material, which reduced the oxidation process of these fatty acids. Taking into account the fatty acid composition, black soldier fly larvae fat is observed to be very similar in composition to coconut oil. Both fats are dominated by lauric acid (C12:0) with up to 50% content of all fatty acids [37,39]. In turn, despite a higher content of SFA, insect fat is characterized by a lower content of palmitic (C16:0), stearic (C18:0), and oleic (C18:1 n-9c) acids compared to, e.g., tallow [40] or lard [41]. But what is worth emphasizing, the presence of lauric acid in insect fat seems to be interesting, as medium-chain fatty acids are preferred in energy utilization compared to long-chain saturated or unsaturated fatty acids. Furthermore, this fatty acid may exhibit antimicrobial effects on gut bacteria [39].

The quality of fat can be evidenced by indicators such as acid value (AV), peroxide value (PV), and oxidative stability [37]. Currently, there are no developed specifications for the quality parameters of fats extracted from edible insects, including those related to oxidative stability. Therefore, it seems reasonable to adopt values such as those in the Codex Alimentarius for edible oils, which are 4 mg KOH/g for the acid value and 15 meq O_2_/kg for the peroxide value.

The acid value is related to the hydrolysis of triacylglycerols (TAGs) and the formation of free fatty acids. Therefore, the acid value is an indicator of the content of free fatty acids in the oil. High levels of free fatty acids are undesirable because they can cause an unpleasant odor and shorten the shelf life of the oil. In order to remove free fatty acids, refining processes are used in industry. The high temperature and high water content will induce thermal hydrolysis, leading to the accumulation of free fatty acids, the levels of which are considered a major quality index of oil [42].

The results of acid value determined in the tested fat samples exceed the value of 4 mg KOH/g. The higher acid value of fat extracted from the IR-CD-dried samples is associated with the release of more free fatty acids formed by the partial hydrolysis of TAGs and the esterification of acylglycerols [37]. This may be related to the higher water content of these samples compared to those dried with the CD method (Figure 1d) as well as presence of endogenous lipases [11]. The lower temperature during the IR-CD drying may not have been sufficient to inactivate lipase enzymes. What is more, the selected parameters of the PEF treatment may activate lipase enzymes and favor the hydrolysis of TAGs.

The peroxide value refers to the primary products of the fat oxidation process. The results of the peroxide value determined in the tested fat samples did not exceed the value of 15 meq O_2_/kg. The higher peroxide value of the fat extracted from the CD-dried samples could be due to reactions between free radicals, induced by PEF treatment, and the present antioxidant compounds (Figure 2). The reaction between them may have caused the formation of peroxide structures, resulting in a higher peroxide value [43]. Furthermore, partial fat decomposition can occur at higher temperatures, which greatly promotes the formation of hydroperoxides. Hurtado-Ribeira et al. [11] conducted a study on the effect of the drying method on the quality of fat from black soldier fly larvae. They demonstrated that fat extracted from convective-dried insects was characterized by a higher peroxide value (4.9 meq O_2_/kg) than that from freeze-dried insects (2.5 meq O_2_/kg). In turn, Matthäus et al. [44] found a lower peroxide value (equal to 0.29 meq O_2_/kg) of fat extracted from black soldier fly larvae dried with a vacuum and then the fluidized-bed method.

Although the primary causes and effects of the oxidation and hydrolysis processes are completely different, scientific research shows that they interact and contribute to shortening the shelf life of the oil. It is assumed that the pro-oxidant effect of free fatty acids is influenced by the carboxyl group, which accelerates the decomposition of hydroperoxides [44]. The higher peroxide value of fat from black soldier fly larvae not subjected to drying could therefore be caused by a higher content of free fatty acids (higher acid value) compared to the dried samples.

The oxidative stability of fat is one of the most important properties. This parameter indicates how susceptible a fat is to the oxidation process and helps determine its shelf life. It may also be useful in indicating the safety of the fat because the oxidation process can result in the formation of harmful products that can adversely affect the health of consumers [37,43]. Oxidative stability is closely related to the content of fatty acids from individual groups, especially unsaturated fatty acids, but also to the presence of antioxidants and pro-oxidant compounds, as well as external factors such as temperature and duration of storage, radiation, and exposure to oxygen and light [11,43]. In general, fat with a higher content of unsaturated fatty acids tends to oxidize faster than fat with a higher content of saturated fatty acids. With regard to fatty acid composition, samples dried with the IR-CD method were characterized by a higher content of saturated fatty acids, resulting in greater oxidative stability. In turn, the main reason for the lower oxidative stability of fat extracted from the CD-dried samples was probably the lower content of bioactive compounds (r = 0.689). The lower oxidative stability may also have been due to the displacement of fatty acids, as a result of increased endogenous lipase activity after PEF application, and the incorporation of unsaturated fatty acids into the external positions of the triacylglycerols, which facilitated the access of oxygen to these acids and their easier oxidation. This is interesting because, at the higher drying temperatures used during convective drying, these enzymes should degrade to a greater extent, which, according to the results of the current study, seems not to be confirmed.

In addition to being a source of compounds such as protein or fat, insects can also provide a new and interesting source of bioactive components [32,45]. A numerous group of bioactive components are polyphenols, which are important in human nutrition. They affect not only the nutritional value of food, but also its antioxidant activity. These bioactive compounds affect the ability to scavenge free radicals, inhibit oxidative stress, and protect against cell damage caused by reactive oxygen species (ROS) [21,30]. However, they are compounds sensitive to various operations and processes that are used during food production [46,47]. In our study, we demonstrated that the total polyphenol content and the antioxidant activity of dried insects are affected by both the intensity of the pulsed electric field and the drying method. The explanations for differences in the results are the drying temperature and the electroporation phenomenon. The higher temperature during the CD method resulted in a greater degradation of polyphenols, which are sensitive to high temperatures [47]. Lucas-Gonzáles et al. [48] demonstrated that freeze-dried house crickets were characterized by higher antioxidant activity than convective-dried ones. In addition, Baek et al. [49] showed the higher antioxidant activity and total polyphenol content of freeze-dried yellow mealworm larvae than of convective-dried. In terms of the PEF effect, changes in tissue structure due to electroporation can be affected in two ways. On the one hand, it affects the leaching of bioactive compounds into the water used as a medium during the PEF pretreatment, since these compounds are soluble in water [46]. On the other hand, the destruction of the structure allows for better penetration of the solvent and greater extractability of the valuable compounds. Furthermore, PEF treatment possibly caused the inactivation of enzymes responsible for various chemical reactions, which may have resulted in a lower demand for antioxidant compounds during the drying of the material [50]. A similar effect of PEF pretreatment on the antioxidant activity of freeze-dried house crickets was noted by Psarianos et al. [51]. The use of PEF with an intensity of 4.9 and 24.5 kJ/kg improved the antioxidant activity of dried insects by up to 58.2% compared to the untreated sample. A decrease in antioxidant activity was observed when the sample was treated at 49.1 kJ/kg. Antioxidant activity is related not only to the presence of bioactive compounds but can also be caused by proteins and peptides. The high positive correlation (r = 0.587) between total polyphenol content and antioxidant activity means that the reduction in polyphenols decreased the antioxidant activity of the dried insect samples. Opposing findings were provided by Pyo et al. [52], who did not find a correlation between these parameters (r = 0.009). Instead, the authors stated that antioxidant activity depends on other compounds such as proteins or other as yet unidentified compounds occurring in insects. As has been reported in the literature, proteins can act as antioxidants and inhibit oxidation reactions due to the presence of low-molecular-weight peptides [32,46,53].

The regulations of the European Union require indication of the presence of allergens on food labels [54]. One of the most crucial allergenic proteins is tropomyosin (TPM). TPM present in the muscles of crustaceans and mollusks, is resistant to digestion by stomach acid and thermal denaturation, leading to a higher likelihood of sensitization [55]. Examples of cross-relationships between crustaceans and edible insect species have been documented [56,57]; however, reports on allergens found in black soldier fly larvae are still limited [58].

Thermal treatment has a limited effect on lowering the content of thermostable allergenic proteins in food [59]. On the other hand, under certain conditions, it may increase the concentration of allergens. Exposure to dry hot air increases the amount of detectable allergenic globular proteins, for instance Ara h1 in peanuts, which undergo covalent cross-linking, hydrophobic interactions, which leads to aggregation to compact polymers with more IgE binding sites [60]. Lamberti et al. [61] reported that hot air and infrared roasting of hazelnuts at 140 °C did not affect the allergenicity of the samples. However, treatment at 170 °C contributed to a reduction in IgE binding, and the reduction was higher for infrared-treated samples. These results are in line with those in the current study.

PEF treatment, depending on the specific energy intensity, can contribute to an increase or decrease in the quantity of detected allergens in the sample. This is due to changes in the structures of secondary and tertiary structures of protein. The study conducted by Yang et al. [62] showed that the ovalbumin (allergenic egg protein) content initially increased with increasing PEF energy (20 and 25 kV/cm), while when a higher intensity (30 and 35 kV/cm) was applied, it decreased. Those observations are consistent with those obtained in the present study.

Thermogravimetric analysis (TGA) is a calorimetric method that can determine the weight loss of a sample at a specific temperature in a variety of foods including powdered foods, fruits, dairy products, and fats, among others. It is a technique that measures the change in weight of a sample when it is heated or held at a constant temperature. In the first stage (30–110 °C), the weight loss of the samples was linked to the evaporation of water and also probably volatile compounds [15]. In the second stage (110–420 °C), the weight loss of the samples was the highest, from 62.8% to 64.2% for the CD-dried samples and from 61.6% to 76.2% for the IR-CD-dried samples. This weight loss in the second stage was connected with the thermal decomposition of organic components: protein and carbohydrates [15,63] and fat, primarily unsaturated fatty acids (PUFA, MUFA), and short-chain saturated fatty acids (SFA) [64]. In the third stage (420–600 °C), the weight loss involved further thermal decomposition of fat—long-chain saturated fatty acids (SFA) [64] and proteins/polypeptides [15,65].

## 4. Materials and Methods

### 4.1. Material

For this research, black soldier fly (*Hermetia illucens* L.) larvae were used. The material was purchased from a local German producer (Ahaus, Germany), and then stored under monitored conditions at a temperature of 4 ± 1 °C until the experiments. For experiments, the material was washed in tap water to remove residual feces and other impurities and then dried with filter paper. The chemical composition and chosen properties of fresh material were presented in Table 5.

### 4.2. Technological Treatment

#### 4.2.1. Pulsed Electric Field Treatment

The pulsed electric field (PEF) treatment was conducted using a pilot-scale PEF unit (PEF Pilot™, Elea GmbH, Quakenbrück, Germany), capable of delivering a 30 kV voltage and monopolar, exponential decay pulses with a frequency of 2 Hz and a pulse width of 7 μs. The gap between the stainless-steel electrodes was 280 mm. Fresh larvae with a mass of approximately 350 g were placed inside the treatment chamber, and tap water (21 ± 1 °C, conductivity of 250 µS/cm) was added until a total weight of 1 kg was achieved. The samples were subjected to an electric field intensity of 1 kV/cm, and the specific energy consumption during the treatment were 5, 20, and 40 kJ/kg [16]. The PEF treatment was performed in duplicate.

#### 4.2.2. Convective Drying

The larvae, both untreated and subjected to PEF treatment, were dried using the prototype laboratory dryer (Warsaw, Poland) with the following parameters: a temperature of 90 °C, an air flow of 2 m/s parallel to the material layer, and a sieve load of 2.71 kg/m^2^. The drying process persisted until the material achieved a state of constant weight and was conducted in duplicate for each sample. The processed material was carefully stored in air and light barrier PET12/AL8/PE100 bags (Pakmar, Warsaw, Poland). Prior to conducting any analyses, the samples were ground to a fine consistency using an analytical mill (IKA A11 basic, IKA-Werke GmbH & Co., Staufen, Germany).

#### 4.2.3. Infrared–Convective Drying

The infrared–convective drying process of untreated and subjected to PEF larvae was performed in prototype laboratory dryer (Warsaw, Poland) with nine lamps positioned 0.25 m from the surface of the dried material and emitting radiation at a level of 7.875 kW/m^2^. The air, heated to the temperature of 40 °C, flowed at the speed of 0.8 m/s parallel to the material layer. Material weighing 2.71 kg/m^2^ was loaded into the dryer. The process was carried out until constant mass was achieved and was conducted in duplicate for each sample. The processed material was carefully stored in air and light barrier PET12/AL8/PE100 bags (Pakmar, Warsaw, Poland). Prior to conducting any analyses, the samples were ground to a fine consistency using an analytical mill (IKA A11 basic, IKA-Werke GmbH & Co., Staufen, Germany).

### 4.3. Characterization of Dried Black Soldier Fly Larvae

#### 4.3.1. Chemical Composition

The fat extraction yield was determined by the Soxhlet method using a Behrotest ET2 Control Unit (Behr Labor-Technik GmbH, Düsseldorf, Germany). The protein content was determined by the Kjeldahl method using a KjelFlex K-360 (Büchi, Flawil, Switzerland) nitrogen analyzer integrated with a TitroLine 5000 automatic titrator (SIAnalytics, Weilheim, Germany). The ash and moisture content were determined by the gravimetric method [33].

#### 4.3.2. Fat Analysis

##### Fatty Acid Composition

Firstly, the fat from the dried insects was extracted using the Folch method [66]. Then, the fatty acids were derivatized into fatty acid methyl esters (FAMEs) using the standard method described in PN-EN ISO 12966:2017 [67]. The YL6100 GC gas chromatograph equipped with a flame ionization detector (FID) and a BPX 70 capillary column (filled with stationary phase, length 60 m, internal diameter 0.25 mm, film thickness 0.25 μm) was used. During the FAMEs separation/analysis, the temperature of the oven was programmed as follows: 70 °C for 5 min, 15 °C/min to 160 °C, 1.1 °C/min to 200 °C, 200 °C for 12 min and 30 °C/min to 225 °C. The injector and detector temperatures were set at 225 °C and 250 °C, respectively. The total time of FAME separation lasted 52 min. The nitrogen was used as a carrier gas and the flow was set at the level of 1 mL/min. The fatty acid content was calculated as an area under the peak in the chromatogram. The relative retention times of the FAME peaks were compared with those of the FAME chemical standard for the identified fatty acids [68]. The analysis was conducted in duplicate for each sample.

##### Oxidative Stability

The oxidative stability of the fat extracted from dried insects was measured by pressure differential scanning calorimetry (PDSC) method using a DSC Q20 TA Instrument (Newcastle, WA, USA) coupled with a high-pressure cell [37]. The analysis was determined at a temperature of 140 °C under an oxygen atmosphere at 1400 kPa in duplicate for each sample.

##### Acid Value

The acid value (AV) of the fat extracted from the dried insects was measured by the titration method with 0.1 mol/L ethanolic potassium hydroxide (KOH) solution using a titrator TitraLab AT100 (HACH LANGE, Wrocław, Poland). The results were expressed as the mg of KOH required to neutralize the acidic constituents present in 1 g of fat [37]. The acid value analysis was conducted in duplicate for each sample.

##### Peroxide Value

The peroxide value (PV) of the fat extracted from dried insects was measured by the titration method with 0.001 mol/L sodium thiosulphate (Na_2_S_2_O_3_) solution using a titrator TitraLab AT100 (HACH LANGE, Wrocław, Poland). The results were expressed as meq O_2_ (milliequivalents of oxygen) per kg of fat [37]. The peroxide value analysis was conducted in duplicate for each sample.

#### 4.3.3. Chemical Analysis

##### Extract Preparation

Two grams of grounded sample were subjected to an extraction process using 10 mL of 80% ethyl alcohol. This extraction was carried out in a dark environment at a controlled temperature of 20 °C for a duration of 12 h, employing a shaker (Multi Reax, Heidolph Instruments, Schwabac, Germany) to facilitate efficient mixing. After the extraction, the solution was subjected to centrifugation at 4350 rpm for 2 min using a laboratory centrifuge (MegaStar 600, VWR, Radnor, PA, USA). Each sample underwent two separate extraction processes to ensure the accuracy and reproducibility of the results.

##### Total Polyphenol Content (TPC)

To quantify the total polyphenol content, a plate reader (Multiskan Sky, Thermo Electron Co., Waltham, MA, USA) was used. A 10 µL portion of the sample extract, along with 10 µL of distilled water and 40 µL of 5-fold diluted Folin–Ciocalteu reagent, were mixed in the well. After 3 min of incubation, 250 µL of supersaturated sodium carbonate was added to the mixture, and the solutions were further incubated at 25 °C for 1 h [69]. The quantitative analysis of total polyphenols was determined by measuring the absorbance of the reaction mixture at a wavelength of 750 nm. A calibration curve, prepared for chlorogenic acid ranging from 0 to 100 µg/mL, was employed for the determination of total polyphenol content. This analysis was conducted in duplicate.

##### Antioxidant Activity (AA)

The antioxidant activity of the dried material was assessed using ABTS^•+^ (2,2′-azino-bis (3-ethylbenzothiazoline-6-sulphonic acid)) free radical solution) [70,71]. A stock solution was prepared 16 h prior to the analysis. This involved dissolving 38.4 mg of ABTS^•+^ powder and 6.6 mg of potassium persulfate in 10 mL of distilled water. On the day of the analysis, a fresh working solution was prepared by diluting the initial mixture approximately 100 times with 80% ethyl alcohol. The absorbance of this working solution was then measured at 734 nm, aiming to fall within the specified range of 0.700 ± 0.020. In the experimental procedure, 10 µL of 20-fold diluted sample extract was combined with 250 µL of the radical working solution in a 96-well plate, and the mixture was incubated at a controlled temperature of 25 °C. After 6 min, the absorbance of the solutions was measured. The degree of radical scavenging, indicative of the antioxidant activity, was determined based on the obtained absorbance readings. To express the results in a standardized manner, the antioxidant capacity was quantified in milligrams of Trolox (a known antioxidant reference compound) per gram of dry substance of the sample.

##### Allergen Content

Allergen content was assessed using the ELISA method. Crustacean and mollusk tropomyosin was determined using the DECRUE01 and DEMOLE01 quantitative test (Demeditec Diagnostics GmbH, Kiel, Germany). About 1 g of powder was dissolved in 20 mL of extraction buffer and incubated at 40 °C for 15 min. The samples were then centrifuged at 2000× *g* for 10 min and the separated supernatant was filtrated. Next, 100 μL of the resulting solution was used per assay according to the manufacturer’s instructions. The absorbance was read at 450 nm (reference wavelength 620 nm), using a BioTek™ 800TS microplate reader (BioTek, Winooski, VT, USA).

#### 4.3.4. Thermal Analysis

Thermal analysis was performed using a thermogravimetric analyzer (TGA/DSC 3+, Mettler Toledo, Greifensee, Switzerland). About 5 mg of the ground sample was placed in open alumina crucibles with a capacity of 70 µL and subjected to pyrolysis at a temperature of 30 to 600 °C. The sample was heated at rate of 5 °C min and under a nitrogen atmosphere with a flow 50 mL/min [72]. TGA and DTG curves were acquired from the differential TGA values using the STAR 16.10 software from Mettler Evaluation. Each sample was done in two replications.

### 4.4. Statistical Analysis

The one-way analysis of variance ANOVA and the post hoc Tukey’s HSD test were applied to assess significant differences between investigated samples using STATISTICA 13.1 (TIBCO Software, Palo Alto, CA, USA). The two-way analysis of variance ANOVA was applied to indicate the significance of the type of treatment (drying method or PEF input energy). For the selected parameters, the data were subjected to Pearson’s correlation analysis to determine the relationship between them. The significance level during the analyses was set at α = 0.05.

## 5. Conclusions

The study aimed to explore how pulsed electric field (PEF) and convective (CD) or infrared–convective (IR-CD) drying techniques affect the chemical composition, fat properties, total polyphenol content, antioxidant properties, allergen content, and thermal properties of black soldier fly larvae. The results showed that both PEF treatment and the drying method significantly affected all the investigated properties of dried insects. The key component of insects is protein. Regardless of the drying method, protein content was the highest in insects after PEF treatment at 5 kJ/kg, while a decrease in its content was observed with a further increase in PEF energy. The utilization of PEF combined with the convective drying method resulted in a higher fat extraction yield and a lower moisture content. What is more, the fat extracted was characterized by a significantly higher MUFA and PUFA content and lower acid values. In turn, the PEF-treated samples that were dried with the IR-CD method were characterized by a higher total polyphenol content and antioxidant activity and a lower allergen content. Furthermore, greater oxidative stability and lower peroxide values were observed for fat extracted from these insects. Changes in total polyphenol content and antioxidant activity were more notable for samples dried with the IR-CD method, obtaining higher values for PEF treated samples. Thus, taking into account all of the results, it can be concluded that samples dried with the IR-CD method allow dried black soldier fly larvae with higher nutritional quality to be obtained. Nevertheless, further studies are required to explain all the changes that occur in the dried insects.

## Figures and Tables

**Table 1:** The results of the effect size of the two-way ANOVA (partial η^2^ and *p*-value).

	PEF Energy		Drying Method		PEF Energy × Drying Method	
	Partial η^2^	*p*-Value	Partial η^2^	*p*-Value	Partial η^2^	*p*-Value
Fat Extraction Yield	0.992	<0.001	0.724	<0.001	0.992	<0.001
Protein Content	0.972	<0.001	0.897	<0.001	0.842	<0.001
Ash Content	0.986	<0.001	0.146	0.118	0.989	<0.001
Moisture Content	0.297	0.121	0.976	<0.001	0.664	<0.001
Saturated Fatty Acids (SFA)	0.907	<0.001	0.979	<0.001	0.955	<0.001
Monounsaturated Fatty Acids (MUFA)	0.836	<0.001	0.969	<0.001	0.890	<0.001
Polyunsaturated Fatty Acids (PUFA)	0.967	<0.001	0.988	<0.001	0.985	<0.001
Acid Value (AV)	0.934	<0.001	0.999	<0.001	0.992	<0.001
Peroxide Value (PV)	0.834	<0.001	0.987	<0.001	0.834	<0.001
Oxidation Stability	0.857	<0.001	0.990	<0.001	0.965	<0.001
Total Polyphenol Content (TPC)	0.965	<0.001	0.987	<0.001	0.947	<0.001
Antioxidant Activity (AA)	0.984	<0.001	0.986	<0.001	0.973	<0.001
Crustacean Content	0.914	<0.001	0.995	<0.001	0.993	<0.001
Mollusk Content	0.957	<0.001	0.811	<0.001	0.977	<0.001

**Table 2:** Fatty acid composition (%) of fat extracted from black soldier fly larvae dried with the convective (CD) and infrared–convective (IR-CD) methods.

	PEF0_CD	PEF5_CD	PEF20_CD	PEF40_CD	PEF0_IR-CD	PEF5_IR-CD	PEF20_IR-CD	PEF40_IR-CD
Capric acid (C10:0)	0.89 ± 0.01 d ^1^	1.18 ± 0.01 e	0.83 ± 0.03 cd	0.88 ± 0.01 d	0.74 ± 0.01 a	0.80 ± 0.03 abc	0.76 ± 0.02 ab	0.82 ± 0.08 bc
Lauric acid (C12:0)	41.56 ± 0.86 a	47.15 ± 0.11 c	41.67 ± 1.25 a	44.85 ± 0.17 b	47.93 ± 0.03 c	44.98 ± 0.40 b	44.40 ± 0.52 b	46.87 ± 1.24 c
Myristic acid (C14:0)	11.90 ± 0.12 a	11.90 ± 0.02 a	11.90 ± 0.04 a	12.01 ± 0.08 a	13.35 ± 0.04 d	12.90 ± 0.09 c	12.55 ± 0.16 b	12.49 ± 0.23 b
Palmitic acid (C16:0)	16.92 ± 0.20 cd	14.72 ± 0.04 a	17.02 ± 0.31 d	16.45 ± 0.11 bc	17.02 ± 0.17 d	17.19 ± 0.16 d	17.14 ± 0.08 d	16.23 ± 0.56 b
Palmitoleic acid (C16:1)	3.12 ± 0.18 d	3.30 ± 0.03 de	3.33 ± 0.06 e	3.29 ± 0.02 de	2.54 ± 0.01 a	2.70 ± 0.04 ab	2.75 ± 0.17 bc	2.91 ± 0.11 c
Margaric acid (C17:0)	0.21 ± 0.04 cd	0.19 ± 0.01 cd	0.21 ± 0.01 d	0.18 ± 0.01 cd	0.11 ± 0.01 a	0.18 ± 0.01 cd	0.17 ± 0.03 bc	0.14 ± 0.02 ab
Stearic acid (C18:0)	3.00 ± 0.13 bc	2.32 ± 0.04 a	3.12 ± 0.13 c	2.93 ± 0.01 b	3.44 ± 0.04 d	3.05 ± 0.04 bc	3.03 ± 0.15 bc	2.89 ± 0.01 b
Oleic acid (C18:1 n-9c)	11.49 ± 0.24 e	9.93 ± 0.03 cd	11.40 ± 0.45 e	10.40 ± 0.01 d	8.26 ± 0.21 a	9.38 ± 0.04 b	9.94 ± 0.28 cd	9.50 ± 0.28 bc
Linoleic acid (C18:2 n-6c)	9.28 ± 0.08 f	7.94 ± 0.03 d	8.84 ± 0.28 e	7.55 ± 0.04 c	5.70 ± 0.01 a	7.53 ± 0.08 c	7.94 ± 0.06 d	6.99 ± 0.15 b
α-Linolenic acid (C18:3 n-3)	0.97 ± 0.06 d	0.84 ± 0.04 c	0.97 ± 0.06 d	0.84 ± 0.01 c	0.50 ± 0.01 a	0.77 ± 0.01 c	0.79 ± 0.06 c	0.67 ± 0.01 b
Arachidic acid (C20:0)	0.54 ± 0.06 cd	0.40 ± 0.01 b	0.57 ± 0.01 d	0.52 ± 0.01 c	0.32 ± 0.01 a	0.42 ± 0.01 b	0.43 ± 0.01 b	0.41 ± 0.04 b
Other acid	0.15 ± 0.01 de	0.15 ± 0.01 de	0.16 ± 0.01 e	0.14 ± 0.01 cde	0.10 ± 0.01 a	0.14 ± 0.01 cd	0.13 ± 0.01 bc	0.12 ± 0.01 b
∑SFA	75.00 ± 0.57 a	77.85 ± 0.01 b	75.31 ± 0.86 a	77.80 ± 0.01 b	82.90 ± 0.21 d	79.51 ± 0.15 c	78.46 ± 0.57 b	79.83 ± 0.55 c
∑MUFA	14.61 ± 0.42 e	13.23 ± 0.06 cd	14.73 ± 0.50 e	13.68 ± 0.03 d	10.80 ± 0.20 a	12.08 ± 0.08 b	12.69 ± 0.45 bc	12.41 ± 0.39 b
∑PUFA	10.24 ± 0.14 f	8.78 ± 0.06 d	9.81 ± 0.34 e	8.38 ± 0.03 c	6.20 ± 0.01 a	8.29 ± 0.08 c	8.73 ± 0.12 d	7.65 ± 0.16 b
n-6/n-3	9.63 ± 0.55 abc	9.52 ± 0.37 abc	9.12 ± 0.24 ab	9.04 ± 0.12 a	11.40 ± 0.35 e	9.84 ± 0.01 bcd	10.07 ± 0.64 cd	10.50 ± 0.11 d

^1^ The different letters within rows indicate significant differences between samples (Tukey’s HSD, α = 0.05).

**Table 3:** Acid value (AV), peroxide value (PV), and oxidative stability of fat extracted from black soldier fly larvae dried with the convective (CD) and infrared–convective (IR-CD) methods.

Sample	Acid Value (mg KOH/g)	Peroxide Value (meq O_2_/kg)	Oxidative Stability (min)
Convective-Dried Black Soldier Fly Larvae			
PEF0_CD	17.38 ± 1.46 a ^1^	1.70 ± 0.05 c	38.34 ± 1.03 a
PEF5_CD	27.01 ± 0.30 b	2.07 ± 0.20 d	44.49 ± 0.80 b
PEF20_CD	32.57 ± 0.84 c	1.16 ± 0.30 b	52.75 ± 1.39 c
PEF40_CD	26.76 ± 1.08 b	1.13 ± 0.22 b	45.79 ± 2.93 b
Infrared–Convective-Dried Black Soldier Fly Larvae			
PEF0_IR-CD	120.59 ± 0.16 f	<0.01 a	84.46 ± 2.93 f
PEF5_IR-CD	114.08 ± 0.78 e	<0.01 a	63.73 ± 5.17 d
PEF20_IR-CD	107.18 ± 0.18 d	<0.01 a	61.65 ± 1.37 d
PEF40_IR-CD	120.55 ± 0.67 f	<0.01 a	76.68 ± 1.22 e

^1^ The different letters within the columns indicate significant differences between the samples (Tukey’s HSD, α = 0.05). The results of the two-way ANOVA are significant (*p* < 0.05).

**Table 4:** Thermal decomposition of black soldier fly larvae dried with the convective (CD) and infrared–convective (IR-CD) methods.

Sample	Step 1		Step 2		Step 3		Sum (%)	Decomposition Temperature (°C)			
	Temp. Range (°C)	Mass Loss (%)	Temp. Range (°C)	Mass Loss (%)	Temp. Range (°C)	Mass Loss (%)					
Convective-Dried Black Soldier Fly Larvae											
PEF0_CD	30–110	0.8	110–420	62.8	420–600	6.5	70.1	–	–	–	358
PEF5_CD	30–110	1.0	110–420	62.9	420–600	6.6	70.5	–	–	–	359
PEF20_CD	30–110	1.1	110–420	64.2	420–600	6.0	71.3	180	–	–	358
PEF40_CD	30–110	1.3	110–420	63.9	420–600	6.8	72.0	186	–	–	357
Infrared–Convective-Dried Black Soldier Fly Larvae											
PEF0_IR-CD	30–110	1.3	110–420	76.2	420–600	5.8	83.2	192	–	339	357
PEF5_IR-CD	30–110	1.4	110–420	61.6	420–600	8.4	71.4	187	305	338	356
PEF20_IR-CD	30–110	1.3	110–420	65.6	420–600	3.5	70.3	189	304	–	351
PEF40_IR-CD	30–110	1.3	110–420	62.5	420–600	1.1	64.9	190	303	338	356

**Table 5:** The chemical composition and selected properties of fresh black soldier fly larvae.

**Properties (Unit)**	**Value (Mean ± SD)**
Moisture content (%)	80.91 ± 2.82
Protein content (g/100 g w.m.)	9.39 ± 0.08
Fat content (g/100 g w.m.)	1.36 ± 0.21
Ash content (g/100 g w.m.)	1.80 ± 0.01
Total polyphenol content (mg ChlA/100 g d.m.)	67.47 ± 0.77
Antioxidant activity (mg TE/g d.m.)	1.83 ± 0.14
Fat properties	
∑SFA	76.89 ± 2.73
∑MUFA	13.91 ± 1.15
∑PUFA	9.00 ± 1.52
n-6/n-3	10.08 ± 2.11
Acid value (mg KOH/g)	178.56 ± 15.32
Peroxide value (meq O_2_/kg)	3.48 ± 3.33
Oxidative stability (min)	22.70 ± 2.88

## Data Availability

The data presented in this study are available on request from the corresponding author.
